# Hospitalization after Cataract Surgery in a Nationwide Managed-Care Population

**DOI:** 10.1371/journal.pone.0149819

**Published:** 2016-02-22

**Authors:** Sophia Y. Wang, Taylor S. Blachley, Chris A. Andrews, John Z. Ayanian, Paul P. Lee, Joshua D. Stein

**Affiliations:** 1 W. K. Kellogg Eye Center, Department of Ophthalmology and Visual Sciences, University of Michigan, Ann Arbor, Michigan, United States of America; 2 Institute for Healthcare Policy and Innovation, University of Michigan, Ann Arbor, Michigan, United States of America; 3 Gerald R. Ford School of Public Policy, University of Michigan, Ann Arbor, Michigan, United States of America; 4 School of Public Health, University of Michigan, Ann Arbor, Michigan, United States of America; 5 Department of Health Management and Policy, University of Michigan School of Public Health, Ann Arbor, Michigan, United States of America; LV Prasad Eye Institute, INDIA

## Abstract

**Purpose:**

Little is known regarding the extent by which patients undergoing outpatient cataract surgery are at risk for postoperative hospitalization. We sought to determine the percentage of patients undergoing cataract surgery who were subsequently hospitalized, the patient characteristics associated with postoperative hospitalization, and the reasons for hospitalization.

**Methods:**

We identified all beneficiaries of a large U.S. managed care network age ≥40 years old who underwent ≥1 cataract surgery from 2001–2011. All enrollees who required inpatient hospitalization within 7, 14, 30, and 90 days following initial cataract surgery and the reasons for hospitalization were determined. Logistic regression was performed to assess factors that significantly impacted the odds of requiring postoperative hospitalization.

**Results:**

Among the 64,981 patients who underwent cataract surgery, rates of hospitalization within 7, 14, 30, and 90 days were 0.3%, 0.5%, 1.3% and 4.2%, respectively. Among the 10,674 patients who had no major preexisting medical comorbidities, 0.1% were hospitalized within 7 days. The odds of hospitalization increased by 35% (OR = 1.35 [CI, 1.23–1.48]) with the presence of each additional comorbidity and by 14% with each additional hospitalization in the 3 years prior to cataract surgery (OR = 1.14 [CI, 1.10–1,18]). Those who were hospitalized in the 30 days prior to cataract surgery had 524% increased odds of being hospitalized within 7 days after cataract surgery (OR = 6.24, [CI, 3.37–11.57]) compared to those with no record of preoperative hospitalization. Postoperative hospitalizations were most commonly due to cardiovascular conditions, comprising over 25% of primary diagnoses associated with hospitalization.

**Conclusions:**

The risk of hospitalization after cataract surgery is low, and is very low among those with no major preexisting medical comorbidities. Opportunities may exist to limit comprehensive preoperative evaluation and testing to those who have serious pre-existing medical comorbidities.

## Introduction

Cataract surgery is the most commonly performed surgery in the United States, with over 2 million operations performed annually[1]. This surgery is considered to be a relatively low-risk procedure[2], often performed in an outpatient setting under monitored anesthesia care[3], General guidelines outline the types of patients who benefit from preoperative physical examinations and diagnostic testing to ensure they are healthy enough to undergo cataract surgery[2,4]. However, the decision whether to perform such testing on a given patient is ultimately made by the surgeon in conjunction with the anesthesiologist. Referrals of Medicare patients for preoperative medical evaluations by family practitioners, general internists, and medical specialists prior to cataract surgery have increased by over 60% from 1998 to 2006[5]. Little is known, however, whether this increased utilization of preoperative evaluations has resulted in improvements in the safety of cataract surgery.

Although there have been several studies describing ocular adverse events following cataract surgery[6,7], little is known regarding the extent to which patients undergoing outpatient cataract surgery are at risk for experiencing serious systemic adverse events requiring inpatient hospitalization during the postoperative period. By identifying the types of medical comorbidities associated with hospitalization following cataract surgery, researchers may be able to develop algorithms that identify patients who are at high risk for requiring hospitalization to focus the necessary resources and preoperative testing on this group of patients. Likewise, if such algorithms also can accurately identify patients who are at low risk for requiring hospitalization during the immediate post-operative period, many such patients may be able to forego such testing with little safety risk. From a societal perspective, there may be opportunities to curtail some of the rapidly rising healthcare costs by only screening those who are most at risk for experiencing difficulties following surgery.

The objective of this study was to determine the percentage of patients undergoing cataract surgery who required subsequent inpatient hospitalization among nearly 65,000 adults in a large United States managed care health plan who underwent cataract surgery during 2001 to 2011. We also identified patient characteristics and comorbid medical conditions associated with postoperative hospitalization and the reasons for hospitalization.

## Methods

### Data Source

The Clinformatics Data Mart database (OptumInsight, Eden Prairie, MN), contains detailed records of managed-care network beneficiaries throughout the United States. We had access to fully de-identified data on all beneficiaries receiving any eye care from January 1, 2001, through December 31, 2011, as identified by at least one International Classification of Diseases (ICD-9-CM)[8] code for any eye-related diagnosis (360–379.9); any Current Procedural Terminology (CPT-4)[9] code for an eye-related visit or diagnostic or therapeutic procedure (65091–68899, 92002–92499); or any claim submitted by an ophthalmologist or optometrist during this period. Medical claims data from inpatient, outpatient, and skilled nursing facilities for all ocular and nonocular conditions were available, as was sociodemographic information on the age, sex, race/ethnicity, education, income, and geographic region of residence of each enrollee. The Clinformatics database has been used in the past to study patients with ocular diseases[10–12].

Because this study used previously collected de-identified data, the University of Michigan Institutional Review Board approved this as a non-regulated study.

### Participants, Sample Selection, and Eligibility

The study population included all persons who underwent at least one cataract surgery in an outpatient setting as identified by Current Procedural Terminology (CPT)[9] codes (66830, 66840, 66850, 66852, 66920, 66930, 66940, 66982–4) and were ≥40 years old at the time of the first cataract surgery. The majority of the cataract surgeries performed were coded as routine with only 3% of all CPT codes for cataract surgery in database for complex cataract surgery (CPT 66982). Persons were required to have been continuously enrolled in the plan for at least 3 years prior to and 90 days after the date of first cataract surgery. Previous studies have demonstrated that compared with medical record documentation, claims data accurately capture patients undergoing cataract surgery[13,14].

### Outcome

The primary outcome of interest was inpatient hospitalization, measured at 7, 14, 30, and 90 days following cataract surgery. If a patient had 1 or more hospitalizations within the follow-up periods of 7, 14, 30, or 90 days after cataract surgery, then they were counted as having been hospitalized during that follow-up period. Inpatient hospitalizations were identified based on admission and discharge dates from the inpatient confinement file in the database. For patients who underwent bilateral cataract surgery (58% of cohort), the date of the first cataract surgery was used. Few patients in the study population (1.3%) had hospitalizations following the second cataract surgery but still within 90 days of the first cataract surgery.

### Data Analysis

All data analyses were performed using SAS software, version 9.3 (SAS Inc., Cary, NC). Characteristics of the study population were summarized using means and standard deviations for continuous variables, and frequencies and percentages for categorical variables.

#### Predictors of hospitalization

Multivariable logistic regression models were used to determine the extent to which sociodemographic factors, certain medical comorbidities, and prior inpatient hospitalization affected the odds of requiring inpatient hospitalization after first cataract surgery. Individual patients were the unit of analysis. Model predictors included age, sex, race/ethnicity, education, income, geographic region of residence, year of surgery, number of inpatient hospitalizations in the 3 years prior to cataract surgery, pre-existing diabetes mellitus and hypertension, congestive heart failure (CHF), cerebrovascular accident (CVA), myocardial infarction (MI), chronic obstructive pulmonary disease (COPD), renal disease, depression, dementia, preoperative electrocardiogram (EKG) testing, and undergoing more than one cataract surgery. Medical comorbidities were identified based on ICD-9-CM codes (Table 1)[8]. In the model, persons with diabetes and hypertension were further categorized as those with “complicated” diabetes or hypertension if they had record of end-organ damage (i.e., nephropathy) or “uncomplicated” if there was no record of end-organ damage. The persons identified with renal disease included those with and without records of treatment with dialysis. In patients who underwent more than one cataract surgery, having a second cataract surgery was considered to be a possible risk factor for hospitalization only if the second surgery occurred before the hospitalization or, if the patient was not hospitalized, if the second surgery occurred within the specified follow-up time period of 7, 14, 30, or 90 days. Preoperative EKGs were those obtained in the 30 days prior to surgery, including the day of surgery.

**Table 1:** ICD-9-CM codes used to identify baseline medical conditions.

Medical Condition	ICD-9-CM codes and code ranges
Diabetes without complications^a^	250.3, 250.30–3, 250.8, 250.80–3, 250.9, 250.90–3, 250.0, 250.00–3, 250.1, 250.10–3, 250.1, 250.20–3
Diabetes with complications^a^	250.4, 250.40–3, 250.5, 250.50–3, 250.6, 250.60–3, 250.7, 250.70–3, 362.01–7
Hypertension without complications^a^	401, 401.0–1, 401.9, 405, 405.0–1, 405.01, 405.09, 405.11, 405.19, 405.9, 405.91, 405.99
Hypertension with complications^a^	362.11, 402, 402.0, 402.00–1, 402.1, 402.10–1, 402.9, 402.90–1, 403, 403.0, 403.00–1, 403.1, 403.10–1, 403.9, 403.90–1, 404.0, 404.00–3, 404.1, 404.10–3, 404.9, 404.90–3
Congestive heart failure	398.91, 402.01, 402.11, 402.91, 404.01, 404.03, 404.11, 404.13, 404.91, 404.93, 425.4–5, 425.7–9, 428, 428.0–2, 428.2, 428.20–3, 428.3, 428.30–3, 428.4, 428.40–3, 428.9
Cerebrovascular accidents	362.34, 430–2,432.0–1, 432.9, 433,433.0–3, 433.8–9, 434, 434.0, 434.00–1, 434.1, 434.10–1, 434.9, 434.90, 434.91, 435, 435.0–3, 435.8–9, 436–7, 437.0–9, 438, 438.0–1, 438.10–2, 438.19, 438.2, 438.20–2, 438.3, 438.30–2, 438.4, 438.40–2, 438.5, 438.50–3, 438.6–8, 438.81–5, 438.89, 438.9
Myocardial infarction	410, 410.0, 410.00–2, 410.1, 410.10–2, 410.2, 410.20–2, 410.3, 410.30–2, 410.4, 410.40–2, 410.5, 410.50–2, 410.6, 410.60–2, 410.7, 410.70–2, 410.8, 410.80–2, 410.9, 410.90–2, 412
Chronic obstructive pulmonary disease / Asthma	416.8, 416.9, 490, 491, 491.0–2, 491.20–2, 491.8–9, 492, 492.0, 492.8, 493, 493.0, 493.00–2, 493.1, 493.10–2, 493.2, 493.20–2, 493.8, 493.81–2, 493.9, 494, 494.0–1, 495, 495.0–9, 496, 500–5, 506.4, 508.1, 508.8
Renal disease	403.01, 403.11, 403.91, 404.02–3, 404.12–3, 404.92–3, 582, 582.0–2,582.4, 582.8, 582.81, 582.89, 582.9, 583.0–2, 583.4, 583.6–7, 585, 585.1–6, 585.9, 586, 588.0, V42.0, V45.1, V56, V56.0–3, V56.31–2, V56.8
Depression	296, 296.0, 296.00–6, 296.1, 296.10–6, 296.2, 296.20–6, 296.3, 296.30–6, 296.4, 296.40–6, 296.5, 296.50–6, 296.6, 296.60–6, 296.7, 296.70–6, 296.8, 296.80–2, 296.89, 296.9, 296.90, 296.99
Dementia	290, 290.0–1,290.1–3, 290.2, 290.20–3, 290.4, 290.40–3, 290.8, 290.9, 294.1, 294.10–1, 331.2

ICD-9-CM = International Classification of Diseases, 9^th^ edition, Clinical Modification

^a^Diabetes and hypertension “with complications” refers to those with evidence of end-organ damage, e.g., nephropathy.

We ran additional models investigating the effect of the timing of preoperative hospitalizations on postoperative hospitalization. In these models, we evaluated whether patients hospitalized within the period of 30 days, 6 months (180 days) and 3 years prior to cataract surgery had increased the odds of being hospitalized postoperatively compared to those with no record of hospitalization before the cataract surgery.

#### Reasons for hospitalization

The Clinformatics database records up to five ICD-9-CM diagnosis codes submitted for each patient encounter. For those who required hospitalization(s) before or after cataract surgery, the primary reason for each hospitalization was identified from the primary (first) ICD-9-CM code listed for the hospitalization encounter. The primary reason for each hospitalization was categorized into more clinically meaningful categories according to the Clinical Classifications Software tool developed for this purpose by the Healthcare Cost and Utilization Project sponsored by Agency for Healthcare Research and Quality[15].

#### Impact of cataract surgery on need for subsequent hospitalization

To compare inpatient hospitalization rates between enrollees who had undergone cataract surgery to other beneficiaries who had been continuously enrolled in the health plan but had not undergone cataract surgery, another analysis was performed in which the study subjects who underwent cataract surgery as described above were matched to controls based on sociodemographic factors and baseline medical comorbidities. Members of the control group were assigned a “sham” cataract surgery date, which was a random date between 3 years after enrolling in the plan and 90 days prior to termination of coverage. Controls were exactly matched to cases based on sex, race/ethnicity, level of education, income, geographic region of residence, urban/rural residence, medical comorbidities, and whether they had been hospitalized in the 3 years prior to cataract surgery or sham cataract surgery date. Matching was also performed for age (at the time of surgery or the sham surgery date) and total number of years in the plan, both of which were matched to within 2 years. Cataract surgery date and sham cataract surgery date were matched to within 1.5 years. Matching was performed in SAS using the %gmatch macro[16], resulting in 39,240 matched pairs (60.4% of the total study population). The proportions of patients hospitalized in the case and control groups within 7, 14, 30, and 90 days of surgery were compared using McNemar’s test.

A sensitivity analysis in which patients who had undergone cataract surgery were matched to controls based on a propensity score calculated from a logistic regression model incorporating the same set of sociodemographic factors and comorbidities as above yielded similar results when hospitalization outcomes were compared; therefore, only the results of matching on each individual factor are presented here.

## Results

### Study sample characteristics

Among the 64,981 eligible beneficiaries who underwent at least one cataract surgery, the mean (SD) length of time in the plan was 7.1 (2.1) years, and the age at first cataract surgery was 68.7 (10.4) years. For those patients who underwent more than one cataract surgery (N = 37672, 58.0% of eligible study patients), the median number of days between the first and second surgery was 28 (interquartile range 14–63 days). There were 57,158 whites (88.0%), 3,091 blacks (4.8%), 2,995 Latinos (4.6%), and 1,277 Asians (2.0%). The study sample included 35,486 women (54.6%); 5,657 enrollees (8.7%) had incomes of > $125,000; and 40,355 enrollees (62.1%) had greater than high school education level. Approximately 23% of those who underwent cataract surgery in our study sample had an EKG obtained in the 30 days prior to surgery. Other characteristics of the cohort are summarized in Table 2.

**Table 2:** Characteristics of the study sample.

N = 64981	**Mean (SD)**
**Age (years)**	68.7 (10.4)
**Number of years in health plan (years)**	7.1 (2.1)
	**N (%)**
**Sex**	
Female	35486 (54.6%)
Male	29495 (45.4%)
**Race/ethnicity**	
White	57158 (88.0%)
Black	3091 (4.8%)
Latino	2995 (4.6%)
Asian	1277 (2.0%)
Other	460 (0.7%)
**Education**	
Less than high school	807 (1.2%)
High school diploma	23819 (36.7%)
Some college	25929 (39.9%)
College	14315 (22.0%)
Advanced degree	111 (0.2%)
**Annual income**	
<$30,000	8123 (12.5%)
$30,000- <60,000	24925 (38.4%)
$60,000- <100,000	19774 (30.4%)
$100,000- <125,000	6502 (10.0%)
≥$125,000	5657 (8.7%)
**United States geographic region**	
Northeast	7468 (11.5%)
West	24748 (38.1%)
South	22196 (34.2%)
Midwest	10524 (16.2%)
Other	45 (0.1%)
**Urban/Rural**	
Urban	56972 (87.7%)
Small rural	4084 (6.3%)
Large rural	3925 (6.0%)
**Cataract surgery date**	
2004–2007	32212 (49.6%)
2008–2011	32769 (50.4%)
**Diabetes**	
Without complications^a^	11284 (17.4%)
With complications	8785 (13.5%)
**Hypertension**	
Without complications	38327 (59.0%)
With complications	9762 (15.0%)
**Congestive heart failure**	9221 (14.2%)
**Cerebrovascular accident**	9197 (14.2%)
**Myocardial infarction**	5230 (8.1%)
**Chronic obstructive pulmonarydisease / Asthma**	18751 (28.9%)
**Renal disease**	5026 (7.8%)
**Depression**	4062 (6.3%)
**Dementia**	1148 (1.8%)
**Underwent preoperative electrocardiogram**	15065 (23.2%)
**Underwent >1 cataract surgery**	37672 (58.0)%

^a^Diabetes and hypertension “with complications” refers to those with evidence of end-organ damage, e.g., nephropathy.

### Hospitalizations Following Cataract Surgery

The percentages of patients admitted to the hospital within 7, 14, 30, and 90 days after initial cataract surgery were 0.3%, 0.5%, 1.3%, and 4.2%, respectively (Table 3). Among the subset of 10,674 persons who had no documented major medical comorbidities prior to undergoing surgery, the proportions hospitalized within 7, 14, 30, and 90 days were roughly one third the rate at each time point (Table 3). By comparison, among those with ≥4 pre-existing medical comorbidities, the proportions hospitalized were roughly three times the average rate in the study population (Table 3).

**Table 3:** Number of patients with hospitalizations before and after cataract surgery based on number of pre-existing comorbidities.

	Total	Within 3 years prior to cataract surgery	Within 6 months prior to cataract surgery	Within 30 days prior to cataract surgery	Within 7 days of cataract surgery	Within 14 days of cataract surgery	Within 30 days of cataract surgery	Within 90 days of cataract surgery
	N (%)	N (%)	N (%)	N (%)	N (%)	N (%)	N (%)	N (%)
**No comorbidities**	10674 (16.4%)	1116 (10.5%)	186 (1.7%)	15 (0.1%)	11 (0.1%)	21 (0.2%)	45 (0.4%)	156 (1.5%)
**1 comorbidity**	19051 (29.3%)	3485 (18.3%)	643 (3.4%)	62 (0.3%)	29 (0.2%)	55 (0.3%)	124 (0.7%)	476 (2.5%)
**2 comorbidities**	17374 (26.7%)	5106 (29.4%)	1034 (6.0%)	92 (0.5%)	31 (0.2%)	65 (0.4%)	177 (1.0%)	604 (3.5%)
**3 comorbidities**	9639 (14.8%)	4409 (45.7%)	996 (10.3%)	85 (0.9%)	38 (0.4%)	74 (0.8%)	173 (1.8%)	540 (5.6%)
**4+ comorbidities**	8243 (12.7%)	5799 (70.35%)	1870 (22.7%)	178 (2.2%)	64 (0.8%)	135 (1.6%)	322 (3.9%)	934 (11.3%)
**All patients**	64981(100%)	19915 (30.7%)	4729 (7.3%)	432 (0.7%)	173 (0.3%)	350 (0.5%)	841 (1.3%)	2710 (4.2%)

### Predictors of hospitalization after cataract surgery

We investigated which factors were predictors of hospitalization within 7, 14, 30, and 90 days after cataract surgery in multivariable logistic regression models (Table 4). Sociodemographic factors besides age were not significantly associated with hospitalization after cataract surgery. For each additional hospitalization in the 3 years prior to cataract surgery, there was a 14% increase in odds of postoperative hospitalization within 7 days (adjusted OR = 1.14 [CI, 1.1–1.18] per hospitalization, P<0.0001). Independent of the number of prior hospitalization, a history of renal disease conferred 56% increased odds of hospitalization within 7 days of surgery (adjusted OR = 1.56 [CI, 1.01–2.40], P = .04), a finding which remained significant at 14, 30, and 90 days following cataract surgery. Undergoing multiple cataract surgeries was not significantly associated with increased postoperative hospitalization within 7 days of cataract surgery, and actually appeared to be protective against hospitalization at 14, 30, and 90 days after cataract surgery. Additional results for the periods 14, 30, and 90 days after cataract surgery are presented in Table 4. As the time period after cataract surgery lengthened, a greater number of comorbidities became significant predictors for hospitalization; nearly all of the examined medical comorbidities were significant predictors for hospitalization at 30 and 90 days after cataract surgery.

**Table 4:** Odds ratios for admission to hospital within 7, 14, 30, and 90 days of cataract surgery based on medical comorbidities.

	Inpatient Hospitalization			
	Within 7 days of cataract surgery	Within 14 days of cataract surgery	Within 30 days of cataract surgery	Within 90 days of cataract surgery
	OR (95% CI)	OR (95% CI)	OR (95% CI)	OR (95% CI)
**Age (per 10-year increase)**	1.02 (0.99–1.03)	**1.02 (1.01–1.04)**	**1.01 (1.01–1.02)**	**1.02 (1.01–1.02)**
**Number of hospitalizations within 3 years prior to surgery (per hospitalization)**	**1.14 (1.1–1.18)**	**1.15 (1.11–1.18)**	**1.16 (1.13–1.20)**	**1.22 (1.20–1.25)**
**Underwent second cataract surgery (vs one cataract surgery)**	0.30 (0.09–0.94)	**0.2 (0.12–0.34)**	**0.33 (0.27–0.41)**	**0.51 (0.46–0.55)**
**Surgery performed 2008–2011 vs 2004–2007**	0.92 (0.67–1.26)	0.87 (0.69–1.09)	0.96 (0.83–1.11)	0.95 (0.87–1.04)
**Diabetes**				
**Without complications**	1.00 (0.66–1.51)	1.03 (0.78–1.37)	1.17 (0.98–1.40)	**1.11 (1.00–1.24)**
**With complications**	1.36 (0.91–2.04)	**1.42 (1.07–1.88)**	**1.33 (1.10–1.61)**	**1.28 (1.15–1.43)**
**Hypertension**				
**Without complications**	1.20 (0.74–1.93)	1.26 (0.90–1.78)	**1.48 (1.18–1.86)**	**1.36 (1.20–1.54)**
**With complications**	1.57 (0.89–2.77)	1.20 (0.79–1.82)	**1.53 (1.16–2.02)**	**1.38 (1.19–1.61)**
**Congestive heart failure**	1.27 (0.85–1.88)	**1.56 (1.19–2.04)**	**1.53 (1.28–1.83)**	**1.42 (1.28–1.58)**
**Cerebrovascular accident**	1.40 (0.98–2.01)	**1.73 (1.35–2.21)**	**1.43 (1.21–1.68)**	**1.32 (1.19–1.45)**
**Myocardial infarction**	1.23 (0.80–1.87)	1.12 (0.83–1.51)	1.20 (0.98–1.45)	1.05 (0.93–1.19)
**Chronic obstructive pulmonary disease / Asthma**	1.34 (0.97–1.85)	**1.58 (1.26–1.99)**	**1.5 (1.29–1.74)**	**1.47 (1.35–1.60)**
**Renal disease**	**1.56 (1.01–2.40)**	**1.43 (1.05–1.95)**	**1.42 (1.15–1.74)**	**1.56 (1.38–1.76)**
**Depression**	1.18 (0.69–2.01)	1.06 (0.72–1.57)	**1.36 (1.07–1.72)**	**1.33 (1.15–1.53)**
**Dementia**	1.59 (0.81–3.13)	1.26 (0.76–2.09)	**1.42 (1.02–1.98)**	**1.26 (1.02–1.55)**
**EKG obtained**	1.10 (0.77–1.55)	1.07 (0.83–1.37)	1.08 (0.92–1.27)	1.00 (0.91–1.09)
**Number of comorbidities**	**1.35 (1.23–1.48)**	**1.40 (1.31–1.49)**	**1.40 (1.34–1.47)**	**1.34 (1.30–1.37)**

The regression models are adjusted for age, sex, race/ethnicity, education, annual income, U. S. geographic region, urban or rural location, year of surgery date, whether the patient underwent more than one surgery, and number of hospitalizations within 3 years prior to surgery. ORs for individual comorbidities are from the same model as and adjusted for other listed comorbidities. OR for number of comorbidities are from a separate model adjusted for sociodemographics, year of surgery date, number of prior hospitalizations within 3 years prior to surgery, electrocardiogram obtained within 30 days prior to hospitalization, but not individual comorbidities. The reference group for all medical conditions is the group with no disease.

Diabetes and hypertension “with complications” refers to those with evidence of end-organ damage, e.g., nephropathy.

Bolded results are significant at P<0.05 level

In a separate logistic regression model adjusted for sociodemographic factors and number of prior hospitalizations, but not for individual comorbidities, we found that the presence of each additional medical comorbidity increased the odds of hospitalization within 7 days of surgery by 35% (adjusted OR = 1.35 [CI,1.23–1.48]), P<0.0001).

We also investigated whether having had ≥1 hospitalizations within certain time periods prior to cataract surgery increased the odds of being hospitalized within 7 days after cataract surgery as compared to those with no record of hospitalization within the 3 years prior to the first cataract surgery. We found that those with a hospitalization in the immediate 30 days prior to surgery had 524% increased odds of being hospitalized within 7 days after cataract surgery (OR = 6.24, [CI, 3.37–11.57], P<0.0001), adjusted for all other covariates. Those with hospitalization within 6 months prior to cataract surgery had 221% increased odds of being hospitalized within 7 days after cataract surgery (OR = 3.21 [CI, 2.24–4.60], P<0.0001). Those with any hospitalizations within 3 years prior to surgery had 176% increased risk of hospitalization within 7 days after cataract surgery (OR = 2.76 [CI, 1.93–3.94], P<0.0001).

### Comparison of hospitalization rates between patients who underwent cataract surgery and matched controls

To investigate whether the low rate of hospitalization after cataract surgery is related to the cataract surgery itself or represents the background risk of hospitalization from the presence of comorbid medical conditions, hospitalization rates of patients who underwent cataract surgery were compared to hospitalization rates of matched controls. Matched pairs in which the case patient was hospitalized within 7 days of cataract surgery but not the control (60 pairs) occurred less frequently than matched pairs in which the control was hospitalized but not the case (108 pairs, P = 0.0003). Additional results for periods 14, 30, and 90 days after cataract surgery are presented in Table 5.

**Table 5:** Difference in hospitalization between patients who underwent cataract surgery and matched controls.

Matched pairs^a^	Within 7 days		Within 14 days		Within 30 days		Within 90 days	
(N = 39240 matched pairs)	N (%)	P^b^	N (%)	P	N (%)	P	N (%)	P
**Discordant**		0.0003		<0.0001		0.002		0.074
Case hospitalized, control not hospitalized	60 (0.2%)		130 (0.3%)		324 (0.8%)		1037 (2.6%)	
Control hospitalized, case not hospitalized	108 (0.3%)		220 (0.6%)		411 (1.0%)		1121 (2.9%)	
**Concordant**								
Both case and control hospitalized	0 (0.0%)		1 (0.0%)		9 (0.0%)		70 (0.2%)	
Neither case nor control hospitalized	39072 (99.6%)		38889 (99.1%)		38496 (98.1%)		37012 (94.3%)	

^a^Cases were subjects who had undergone cataract surgery. Cases and controls were matched on age +/- 2 years, length of time enrolled in the health plan +/- 2 years, sex, race/ethnicity, education, income, geographic region, and urban/rural setting, presence of hospitalization in the 3 years prior to cataract surgery or sham cataract surgery date, baseline diabetes, congestive heart failure, cerebrovascular accidents, myocardial infarction, chronic obstructive pulmonary disease, renal disease, mood disorders, and dementia.

^b^Exact version of McNemar’s test used to determine P value.

### Reasons for hospitalization after cataract surgery

Among those who were admitted to the hospital within 7, 14, 30, and 90 days following cataract surgery, cardiovascular conditions were the most common reason for the hospitalization, encompassing >25% of admission diagnoses, with CHF and hypertension among the most common diagnoses in this category (Table 6). Hospitalizations for COPD, pneumonia, and urinary tract infections were also common. Hospitalizations specifically related to ophthalmologic complications were uncommon (≤1.1% of hospitalizations), and included one hospitalization for purulent endophthalmitis within 7 days of the cataract surgery. The most common reasons for hospitalization in the 3 years prior to surgery were very similar to reasons for hospitalization subsequent to surgery, with cardiovascular diagnoses a main reason for admission as well as COPD and infections.

**Table 6:** Diagnoses for hospitalization events after cataract surgery.

	Within 3 years prior to surgery	Within 7 days after surgery	Within 14 days after surgery	Within 30 days after surgery	Within 90 days after surgery
	(N = 38,736)	(N = 175)	(N = 365)	(N = 922)	(N = 3317)
	N (%)	N (%)	N (%)	N (%)	N (%)
**Cardiovascular**	**10,167 (26.2%)**	**47 (26.9%)**	**105 (28.8%)**	**263 (28.5%)**	**847 (25.5%)**
Hypertension	2331 (6.0%)	13 (7.4%)	28 (7.7%)	76 (8.2%)	235 (7.1%)
Congestive heart failure	1283 (3.3%)	8 (4.6%)	18 (4.9%)	42 (4.6%)	134 (4.0%)
Dysrhythmias and conduction disorders	1641 (4.2%)	7 (4.0%)	17 (4.7%)	47 (5.1%)	174 (5.3%)
Chest pain and acute coronary syndromes	3375 (8.7%)	11 (6.3%)	26 (7.1%)	61 (6.6%)	174 (5.3%)
Cardiac arrest and ventricular fibrillation	34 (0.1%)	1 (0.6%)	1 (0.3%)	3 (0.3%)	6 (0.2%)
Other cardiac and vascular diagnoses	1503 (3.9%)	7 (4.0%)	15 (4.1%)	34 (3.7%)	124 (3.7%)
**Pulmonary**	**3154 (8.1%)**	**20 (11.4%)**	**46 (12.6%)**	**114 (11.1%)**	**336 (10.1%)**
Chronic obstructive pulmonary disease or asthma	1756 (4.5%)	12 (6.9%)	24 (6.6%)	59 (6.4%)	184 (5.6%)
Other upper and lower respiratory tract disorders	1398 (3.6%)	8 (4.6%)	22 (6.0%)	43 (4.7%)	152 (4.6%)
**Infectious diseases**	**3253 (8.4%)**	**15 (8.6%)**	**32 (8.8%)**	**78 (8.5%)**	**320 (9.6%)**
Pneumonia	851 (2.2%)	7 (4.0%)	9 (2.5%)	23 (2.5%)	99 (3.0%)
Urinary tract infection	637 (1.6%)	5 (2.9%)	7 (1.9%)	20 (2.2%)	70 (2.1%)
Other infections (non-ocular)	1765 (4.6%)	3 (2.3%)	16 (4.4%)	35 (3.8%)	151 (4.6%)
**Gastroenterology or hepatobiliary**	**3108 (8.0%)**	**17 (9.7%)**	**33 (9.0%)**	**68 (6.5%)**	**199 (6.0%)**
**Renal, genitourinary, or electrolyte disturbances**	**3354 (8.7%)**	**15 (8.6%)**	**26 (7.1%)**	**72 (7.8%)**	**330 (10.0%)**
**Oncology/Hematology**	**2066 (5.3%)**	**9 (5.1%)**	**20 (5.5%)**	**55 (6.0%)**	**205 (6.2%)**
**Falls, fractures, injuries, wounds, musculoskeletal or skin disorders**	**3608 (9.3%)**	**11 (6.3%)**	**19 (5.2%)**	**58 (6.3%)**	**223 (6.7%)**
**Endocrine**	**1524 (3.9%)**	**11 (6.3%)**	**16 (4.4%)**	**43 (4.7%)**	**144 (4.3%)**
Diabetes	1129 (2.9%)	9 (5.1%)	13 (3.6%)	33 (3.6%)	110 (3.3%)
Other endocrine disorders	395 (1.0%)	2 (1.1%)	3 (0.8%)	10 (1.1%)	34 (1.0%)
**Neurologic**	**1738 (4.5%)**	**7 (4.0%)**	**15 (4.1%)**	**38 (4.1%)**	**144 (4.3%)**
Stroke, transient ischemic attacks, and other cerebrovascular disease	737 (1.9%)	3 (1.7%)	4 (1.1%)	6 (0.7%)	35 (1.1%)
Delirium or dementia	103 (0.3%)	0 (0.0%)	2 (0.6%)	7 (0.8%)	16 (0.6%)
Other neurologic disorders	898 (2.3%)	4 (2.3%)	9 (2.5%)	25 (2.7%)	93 (2.8%)
**Psychiatric and substance use**	**724 (1.9%)**	**1 (0.6%)**	**5 (1.4%)**	**16 (1.7%)**	**66 (2.0%)**
**Rheumatologic**	**190 (0.5%)**	**1 (0.6%)**	**1 (0.3%)**	**6 (0.7%)**	**20 (0.6%)**
**Gynecologic**	**353 (0.9%)**	**0 (0.0%)**	**0 (0.0%)**	**4 (0.4%)**	**11 (0.3%)**
**Complications of devices, grafts, transplants, procedures, and medical care**	**1221 (3.2%)**	**5 (2.9%)**	**12 (3.3%)**	**26 (2.8%)**	**87 (2.6%)**
**Ophthalmologic**	**121 (0.0%)**	**2 (1.1%)**	**3 (0.8%)**	**4 (0.4%)**	**10 (0.3%)**
Eye infection	16 (0.0%)	1 (0.6%)	1 (0.3%)	1 (0.1%)	1 (0.0%)
Cataract	6 (0.0%)	0 (0.0%)	0 (0.0%)	0 (0.0%)	1 (0.0%)
Glaucoma	19 (0.1%)	0 (0.0%)	0 (0.0%)	1 (0.1%)	3 (0.1%)
Retinal disease	41 (0.1%)	0 (0.0%)	0 (0.0%)	0 (0.0%)	1 (0.0%)
Blindness or visual impairment	23 (0.1%)	0 (0.0%)	0 (0.0%)	0 (0.0%)	0 (0.0%)
Myopia	0 (0.0%)	1 (0.6%)	1 (0.3%)	1 (0.1%)	1 (0.0%)
Other eye disease	16 (0.0%)	0 (0.0%)	1 (0.3%)	1 (0.1%)	3 (0.1%)
**Miscellaneous**	**2639 (6.8%)**	**6 (3.4%)**	**15 (4.1%)**	**55 (6.0%)**	**205 (6.2%)**
**Missing diagnosis**	**1516 (3.9%)**	**8 (4.6%)**	**17 (4.7%)**	**44 (4.8%)**	**170 (5.1%)**

## Discussion

Hospitalization after cataract surgery was uncommon in this patient population, and even rarer among those who had no major pre-existing medical comorbidities or prior hospitalizations. Only 3 of every 1000 patients overall were hospitalized within 7 days of cataract surgery, and only 1 of every 1000 of patients without major medical comorbidities were hospitalized within 7 days of surgery. The major predictors of hospitalization after cataract surgery, particularly in the immediate postoperative period, were a history of prior hospitalizations and co-existing renal disease. In fact, the more recently the patient had been hospitalized prior to cataract surgery, the higher the odds of hospitalization immediately after cataract surgery, with over 5-fold increase in the odds of hospitalization if the patient had been hospitalized in the month prior to surgery. The odds of requiring hospitalization post-operatively increased with the number comorbid conditions, and the most common diagnoses associated with hospitalizations were cardiac in nature.

Previous literature has also suggested that prior inpatient hospitalization is a risk factor for adverse events after cataract surgery, even while cataract surgery has been shown to be relatively safe overall. A study of a large Veteran’s Health Administration (VA) population showed that prior hospital admissions were associated with increased mortality within 90 days of cataract surgery, as were the comorbidities of COPD and CHF[17]. A large study of Medicare beneficiaries also found that prior hospitalization was associated with increased odds of emergency department visits or hospitalization within 7 days of cataract surgery[18]. The same study found that of 16 different outpatient surgeries, cataract surgery was the least associated with a subsequent emergency department visit or hospitalization within 7 days[18]. In a multicenter study of over 19,000 cataract surgeries, Katz and colleagues found the majority of diagnoses associated with death or hospitalization within 7 days of cataract surgery were cardiovascular, similar to our results. They also reported a very low 0.3% rate of postoperative death or hospitalization within 7 days[3]. Our study builds on previous studies by showing that hospitalizations that are closer in timing to the date of the cataract surgery are associated with a much higher odds of postoperative hospitalization, and by additionally identifying comorbid renal disease as a risk factor for hospitalization after cataract surgery, as well as the total number of comorbidities overall. Furthermore, our study presents findings from more recent surgeries performed in the last decade, as well as from a large population of managed care network beneficiaries which may be more widely representative than patients from the VA, covered Medicare, or undergoing surgery at selected centers.

Previous studies have demonstrated that routine preoperative testing, such as obtaining a complete blood count, chemistry panel, or electrocardiogram do not lead to improved safety or outcomes following cataract surgery[19–22]. The Joint Commission on Accreditation of Healthcare Organizations has no specific mandates for routine diagnostic testing[23] and guidelines from the American College of Cardiology/American Heart Association and the Institute for Clinical Systems Improvement suggest that routine preoperative testing such as with EKGs is not warranted for cataract surgery[2,24], even for persons age 65 years and older[24]. Furthermore, the Centers for Medicare and Medicaid Services (CMS) does not cover routine preoperative EKGs or age-based EKG screening. Reimbursement for EKG testing requires documentation of signs and symptoms demonstrating an indication for such testing[23]. However, data from 1995 found that 70 to 90% of ophthalmologists have reported frequently or always obtaining a complete blood count, electrolytes, and EKG in patients being considered for cataract surgery and who had no history of major medical problems[25] and in the present analysis, nearly one quarter of patients who underwent cataract surgery had a record of one of more EKG test in the month leading up to the surgery. Furthermore, referrals for preoperative consultations by family practitioners, general internists, and specialists for Medicare patients undergoing cataract surgery have increased 60% in the period from 1995 to 2006, even after differences in patient characteristics were accounted for[5]. Since previous studies have demonstrated that routine preoperative testing, such as complete blood count, chemistry panels, or EKG does not lead to improved safety or outcomes[19–22], preoperative exams and tests may represent the utilization of resources, such as physician time and costs of tests, with unclear benefit. Considering we found in our study that patients with no previous comorbidities or prior hospitalizations in the 3 years leading up to surgery had exceedingly low rates of postoperative hospitalization, these patients may be especially unlikely to benefit from routine preoperative testing. Those with serious pre-existing medical comorbidities such as renal disease or a history of a recent hospitalization may be more likely to benefit from preoperative evaluation or testing.

A major strength of this study was the inclusion of a large and diverse population seen by healthcare providers in multiple settings and geographic regions, which allows the study to be generalizable to a wide population of insured U.S. residents. Furthermore, information from this large managed-care health insurance network over a decade of time provided the ability to capture and study rare events such as hospitalization after cataract surgery. In addition, the dataset offers detailed and meaningful characterization of these events, such as the diagnoses associated with the hospitalization and comorbidities associated with the hospitalization. Finally, the large study population allowed us to exactly match cases who underwent cataract surgery with controls who did not undergo cataract surgery on a wide range of sociodemographic characteristics and medical comorbidities which otherwise would not be feasible in a smaller sample.

The nature of healthcare claims data also placed several limitations on our study. Results may not be applicable to those without health insurance. Intraoperative and preoperative clinical data were unavailable, such as blood pressure, blood sugar, or creatinine at the time of surgery. There is no way to determine with certainty whether hospitalization could be attributed to the cataract surgery itself or other related factors such as anesthesia or patient-related factors. Indeed, many additional comorbidities became significant predictors of hospitalization at 30–90 days likely because hospitalization at those time periods was more a consequence of comorbid illness than of the cataract surgery itself, especially given our findings that matched controls were just as likely or more likely to be hospitalized within the same time period.

The observational nature of the study exposes it to selection and surveillance biases which are further limitations to interpretation of the results. There was no way to identify patients who underwent and failed preoperative screening tests and therefore did not have cataract surgery. Prior studies have suggested that cancellation of cataract surgery is only approximately 2% regardless of whether or not preoperative screening tests are performed[19,22]. The possibility remains that those who underwent cataract surgery may have been healthier than the total number of patients who were considered for surgery.

In summary, the risk of hospitalization after cataract surgery is low, and is very low among those with no major pre-existing medical comorbidities. The number of prior hospitalizations and comorbid renal disease were found to be important risk factors for hospitalization soon after cataract surgery. A recent hospitalization within the month prior to surgery was a particularly strong risk factor for postoperative hospitalization. Opportunities may exist to reduce overall healthcare costs without adversely impacting patient safety by limiting comprehensive preoperative evaluation and testing for patients considering cataract surgery to those who have serious pre-existing medical comorbidities or recent prior hospitalizations.
